# CFD-Based Aerodynamic Characterization of the Air-Sampling Subsystem of a Portable Radioactive Aerosol Monitoring System

**DOI:** 10.3390/s26206367

**Published:** 2026-10-09

**Authors:** Natalia Alegría, Igor Peñalva

**Affiliations:** Energy Engineering Department, University of the Basque Country, 48013 Bilbao, Spain; igor.penalva@ehu.eus

**Keywords:** radioactive aerosol, radiation detection, radiation monitoring, aerosol sampling, air sampling, portable sensor, CeBr_3_ detector, computational fluid dynamics, porous medium, pleated filter, MARE system, environmental radiation monitoring

## Abstract

Portable radioactive aerosol monitoring systems integrate air sampling, aerosol collection, and radiation detection within a single measurement chain. In such systems, the aerodynamic conditions established during air transport through the collection filter may influence the spatial distribution of the sampled aerosol and therefore represent an important aspect of system characterization. The portable Monitoring Air pump for Radioactive Aerosol (MARE) system incorporates a high-flow air pump, an airflow monitoring unit, a pleated H13 HEPA aerosol collection filter, and a CeBr_3_ scintillation detector for the continuous monitoring of airborne radioactive aerosols. In the present study, computational fluid dynamics (CFD) was employed to characterize the aerodynamic behaviour of the MARE air-sampling subsystem. Two complementary numerical models were developed using STAR-CCM+: a three-dimensional (3D) model representing the complete sampling configuration and a two-dimensional (2D) model representing a characteristic section of an individual filter pleat. The H13 HEPA filter was represented as a homogeneous porous medium using a Darcy–Forchheimer resistance formulation, thereby accounting for its macroscopic hydraulic resistance without explicitly resolving the underlying fibre-scale structure. The 3D model predicted a pressure drop of 268.7 Pa across the filter, while the corresponding 2D representative-pleat model yielded a pressure drop of 262.3 Pa. The resulting difference of approximately 2.4% indicates a consistent global hydraulic response between the two modelling approaches under the investigated operating condition. However, the 3D simulation revealed a pronounced spatial non-uniformity in the mass-flux distribution over the filter surface. The circumferential distribution was comparatively uniform at a given elevation, whereas the dominant variation occurred along the vertical direction, with higher mass flux predicted in the lower region of the filter, where the influence of the downstream suction system was strongest. At the individual-pleat scale, the mean mass flux was 1.36444 kg m^−2^ s^−1^. The internal region of the representative pleat exhibited mass-flux values approximately 30% above the mean, whereas the external region showed values approximately 10% below the mean, demonstrating substantial local redistribution of airflow within the pleated geometry. These results demonstrate that global pressure-drop measurements alone are insufficient to fully characterize the spatially resolved aerodynamic conditions within the sampling stage. CFD provides complementary information by resolving the local airflow distribution and identifying systematic flow non-uniformities that may be relevant to subsequent investigations of aerosol transport and deposition. The present results therefore provide an aerodynamic basis for future particle-transport and deposition studies aimed at establishing the relationship between local sampling conditions, aerosol collection, and the subsequent radiological response of the MARE system.

## 1. Introduction

Portable radioactive aerosol monitoring systems integrate air sampling, aerosol collection, and radiation detection within a single measurement chain. In these systems, ambient air is actively transported through a sampling path and a collection filter, where airborne particulate matter is retained and the associated radioactive material is subsequently detected. The measurement process therefore comprises a sequence of coupled stages, including air intake, flow transport, aerosol collection, accumulation of radioactive material, and radiation detection. The aerodynamic conditions established within the sampling subsystem constitute a fundamental component of this chain because they determine the spatial distribution of airflow over the collection surface and define the transport conditions experienced by aerosol particles prior to their retention by the filter.

This consideration is particularly relevant to high-flow sampling systems employing pleated high-efficiency particulate air (HEPA) filters. Compared with a flat filter having the same external footprint, a pleated configuration provides a substantially larger effective filtration area within a compact volume. However, the three-dimensional pleat geometry, together with the configuration of the surrounding flow path and downstream extraction system, can generate spatially non-uniform airflow through the filter. Such non-uniformity cannot be adequately characterized using global parameters such as volumetric flow rate or total pressure drop alone. A spatially resolved characterization of the flow field is therefore required to identify local variations in filtration conditions and to determine the extent to which the nominal average operating conditions represent the actual aerodynamic environment across the collection surface.

The Monitoring Air pump for Radioactive Aerosol (MARE) system is a portable instrument designed for the continuous monitoring of airborne radioactive aerosols. The system integrates a high-flow air pump, an airflow monitoring unit, a pleated H13 HEPA aerosol collection filter, a cerium bromide (CeBr_3_) scintillation detector, digital pulse-processing electronics, and a central control and data-acquisition unit. The sampling subsystem operates at flow rates of up to 200 m^3^ h^−1^. The pleated filter surrounds the detector assembly, thereby providing a large aerosol collection area within the constrained volume of the instrument. The combination of high-volume air sampling and an internally positioned radiation detector makes the aerodynamic characterization of the sampling path particularly relevant to the overall performance and interpretation of the measurement system.

Recent developments in aerosol monitoring have increasingly emphasized compact, portable, and remotely connected instrumentation capable of providing spatially and temporally resolved information on airborne contaminants. In atmospheric aerosol characterization, complementary measurement techniques, including aethalometers, micro-pulse lidar, and portable optical particle profilers, have been investigated for the retrieval of near-surface aerosol properties [1]. Portable air-quality monitoring systems based on cellular Internet-of-Things (IoT) technologies have also been developed for outdoor pollution mapping [2], while standardized sampling strategies have been proposed for the monitoring of volatile organic compounds in indoor environments [3]. These developments reflect a broader trend towards distributed air-monitoring systems in which compact instrumentation, continuous sampling, data acquisition, and remote operation are increasingly integrated.

Comparable requirements arise in radiological applications, where the early detection of airborne radioactive contamination requires reliable sampling and detection systems capable of continuous operation under potentially variable environmental conditions. Compact radioactive aerosol monitoring devices have been developed for early-warning networks [4], while rapidly deployable spectrometric air-sampling systems have been investigated and characterized using Monte Carlo methods [5]. More recent studies have addressed portable radioactive aerosol monitoring prototypes [6], portable aerosol monitors incorporating signal-processing and linear-deconvolution methods for the rapid identification of alpha emitters [7], and remotely controlled continuous aerosol-monitoring systems for environments with elevated radon concentrations [8]. Collectively, these studies demonstrate the increasing relevance of compact, portable, and remotely operable platforms for aerosol and radioactive aerosol monitoring. They also highlight the importance of understanding the physical processes governing air sampling and aerosol transport within compact monitoring systems.

From a physical modelling perspective, the airflow field represents one stage of the complete radioactive aerosol measurement process. The present study is restricted to the continuous gas phase and does not explicitly resolve the trajectories or interactions of individual aerosol particles. Consequently, the CFD model does not directly quantify particle deposition, particle-size-dependent filtration efficiency, aerosol collection efficiency, spatial distribution of accumulated radioactive activity, radiation transport, or detector response. Although the calculated airflow field establishes the aerodynamic conditions under which aerosol particles are transported through the sampling system, establishing a quantitative relationship between local airflow, particle deposition, accumulated radioactive activity, and detector response requires additional particle-transport, deposition, and radiation-transport analyses and/or experimental measurements. These processes are therefore beyond the scope of the present investigation.

Computational fluid dynamics (CFD) provides an appropriate framework for characterizing the aerodynamic behaviour of complex sampling configurations and for resolving spatial variations that cannot be obtained from global pressure-drop measurements alone. Spatially resolved numerical modelling is particularly relevant for systems in which local flow conditions may differ substantially from nominal average values. Recent developments in computational modelling have demonstrated the capability of advanced numerical approaches to resolve complex flow and multiphysics phenomena and to support system-level assessment and predictive modelling [9,10]. Although these methodologies have been developed in different engineering contexts, they reinforce the value of spatially resolved computational approaches for systems in which local physical conditions may influence overall performance.

In the present work, the H13 HEPA filter is represented as a homogeneous porous medium using a Darcy–Forchheimer resistance formulation. The individual filter fibres and pore-scale structure are not explicitly resolved; instead, their collective hydraulic effect is represented through effective viscous and inertial resistance coefficients. This homogenized representation enables the macroscopic pressure loss of the filter to be incorporated into the system-level CFD model while maintaining a computationally tractable representation of the complete sampling configuration. Accordingly, the numerical analysis focuses on the pressure loss across the filter and on the spatial distribution of airflow and mass flux over its pleated geometry.

The objective of the present study is to numerically characterize the aerodynamic behaviour of the MARE air-sampling subsystem and to determine the spatial distribution of airflow through the pleated H13 HEPA filter under the nominal operating condition. Two complementary CFD models are employed to investigate the flow at different spatial scales. A three-dimensional (3D) model represents the complete sampling configuration and is used to characterize the system-level pressure and airflow fields, including circumferential and vertical variations in mass flux. A two-dimensional (2D) model of a representative filter section is subsequently employed to investigate local mass-flux redistribution within an individual pleat.

The specific objectives of the study are:

- To quantify the pressure drop across the pleated H13 HEPA filter of the MARE air-sampling subsystem;
- To characterize the airflow pattern within the complete sampling configuration;
- To determine the spatial distribution of mass flux over the filter surface;
- To evaluate circumferential and vertical variations in mass flux;
- To characterize the local flow distribution within a representative filter pleat;
- To assess the implications and limitations of the predicted aerodynamic conditions for subsequent characterization of the complete radioactive aerosol monitoring system.

Accordingly, the scope of the present work is limited to the aerodynamic characterization of the MARE air-sampling subsystem under steady, single-phase airflow conditions. The results are not intended to provide a direct assessment of aerosol deposition, particle collection efficiency, radioactive activity distribution, or radiation detector performance. Rather, the study establishes the spatially resolved aerodynamic conditions within the sampling path and identifies systematic flow non-uniformities that may be relevant to subsequent investigations of aerosol transport, deposition, and radiation detection.

The principal contribution of this work is the spatial characterization of the aerodynamic behaviour of the air-sampling subsystem of a portable radioactive aerosol monitoring system using complementary CFD models at system and pleat scales. The analysis demonstrates that global pressure-drop information alone does not fully describe the local flow conditions established over a pleated filter. The resulting aerodynamic characterization provides a quantitative basis for subsequent investigations incorporating Lagrangian aerosol-particle transport, deposition modelling, and radiation-transport and detector-response analyses, thereby supporting a more comprehensive characterization of the complete radioactive aerosol measurement chain.

## 2. Materials and Methods

### 2.1. MARE System

**Figure 1:**
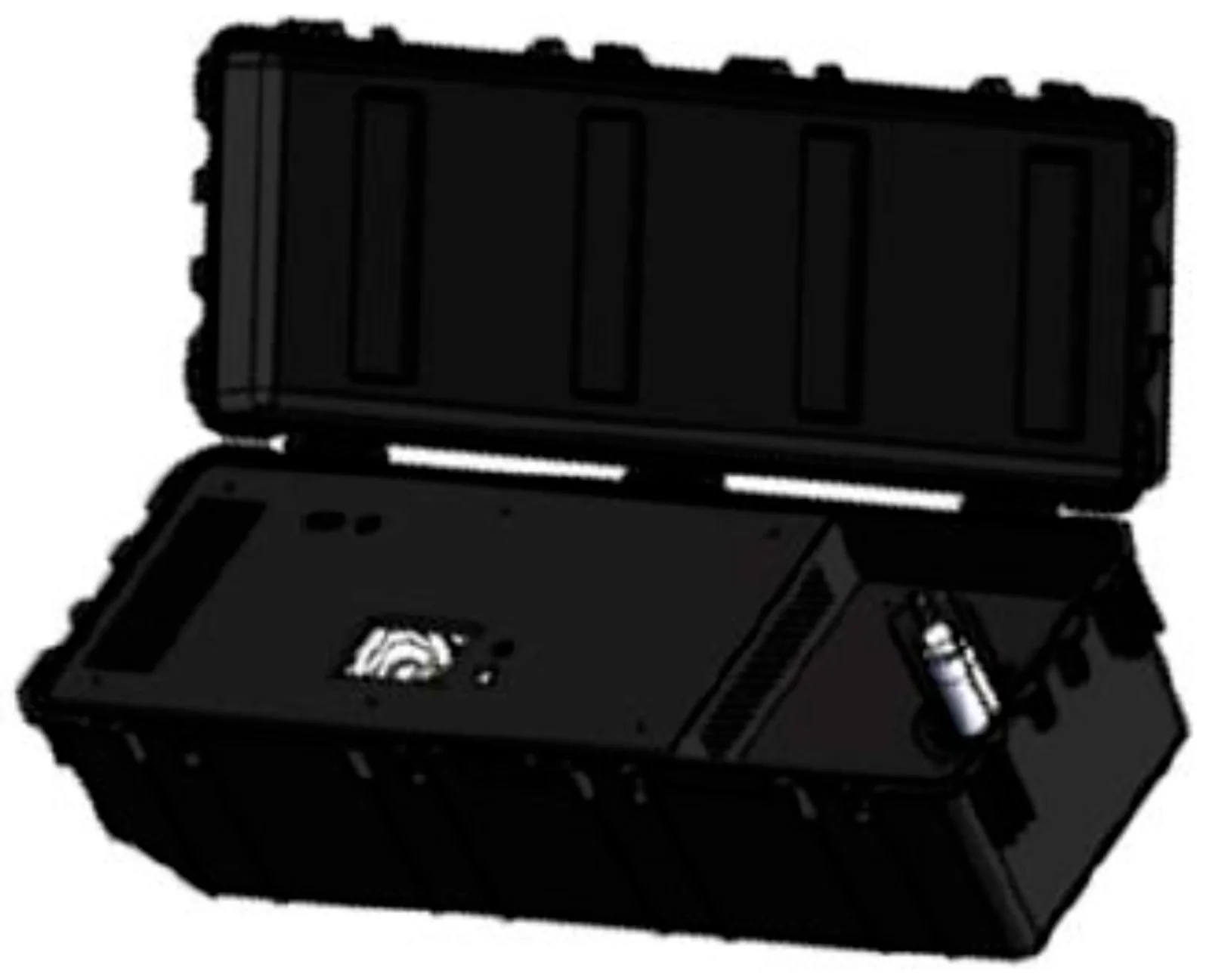
MARE portable radioactive aerosol monitoring system. The instrument integrates the high-flow air pump, airflow monitoring unit, pleated H13 HEPA aerosol collection filter, internal CeBr_3_ scintillation detector, digital pulse-processing electronics, and central control and data-acquisition system.

The Monitoring Air pump for Radioactive Aerosol (MARE) system, shown in Figure 1, is a portable instrument designed for the continuous monitoring of radioactive aerosols in air [11,12]. The system integrates air sampling, aerosol collection, and gamma-ray detection within a single compact platform, enabling the near-real-time characterization of airborne radioactive contamination under field-deployment conditions.

The system architecture comprises the following main subsystems:

- High-flow air pump;
- Airflow monitoring unit;
- Pleated H13 HEPA aerosol collection filter;
- Cerium bromide (CeBr_3_) scintillation detector;
- Digital pulse-processing electronics;
- Central control and data acquisition unit.

All components are housed within a Peli Case 1740 enclosure, which provides mechanical protection while maintaining the portability required for field operation. The air-sampling subsystem is designed to operate at flow rates of up to 200 m^3^·h^−1^, providing a high sampling throughput suitable for the detection of low concentrations of airborne radioactive material.

The measurement process is initiated by drawing ambient air through the sampling subsystem and directing it towards the pleated H13 HEPA collection filter. As the sampled air passes through the filter medium, suspended aerosol particles are captured by the filter. Consequently, radioactive material associated with the airborne particulate fraction is accumulated on the collection surface, producing a localized activity distribution that can subsequently be characterized by gamma-ray spectrometry.

The radiation-detection subsystem consists of a cylindrical CeBr_3_ scintillation detector positioned within the filter assembly. This configuration provides direct geometrical coupling between the accumulated radioactive material and the detector, thereby enhancing the detection efficiency for gamma radiation emitted from the collected activity. The scintillation signal is digitized using a 14-bit analogue-to-digital converter (ADC) and subsequently processed by a field-programmable gate array (FPGA)-based digital pulse-processing system. The resulting energy spectrum comprises 4096 channels, providing sufficient spectral resolution for the identification and quantitative assessment of gamma-emitting radionuclides.

The overall measurement chain can therefore be described as a sequential process comprising:

Ambient air → Air sampling → Aerosol collection → Accumulated radioactive material → CeBr_3_ detection → Signal processing

The integration of high-volume air sampling with in situ gamma-ray spectrometry enables the MARE system to operate as a compact and autonomous platform for continuous or near-real-time monitoring of radioactive aerosols. From an aerodynamic perspective, the high sampling flow rate and the three-dimensional geometry of the pleated filter are particularly relevant, as they determine the distribution of airflow over the collection surface and, consequently, the transport conditions experienced by airborne particulate matter within the sampling subsystem. These characteristics provide the basis for the computational fluid dynamics analysis presented in the following sections.

### 2.2. Filter Configuration

The aerosol collection stage consists of a pleated, concertina-type filter surrounding the detector assembly. The filter comprises 111 pleats, providing a large effective filtration area within the constrained volume of the monitoring system. This configuration increases the available collection surface relative to a planar filter with the same external footprint and enables high volumetric sampling rates while maintaining relatively low average filtration velocities.

According to the technical documentation considered in this study, the collection medium is classified as an H13 high-efficiency particulate air (HEPA) filter. Under the operating conditions considered, the expected initial pressure drop across the filter is in the range of 250–300 Pa [13,14]. The hydraulic behaviour of HEPA filters, including the dependence of pressure drop on filtration velocity and operating conditions, has been extensively investigated [15]. The H13 classification provides high removal efficiency for airborne particulate matter while maintaining a pressure loss compatible with high-flow-rate operation.

The pleated configuration substantially increases the effective filtration area compared with an equivalent flat filter. For a given volumetric flow rate, this increase in area reduces the nominal filtration velocity and consequently contributes to maintaining an acceptable pressure drop and filtration performance. However, the three-dimensional geometry of the pleated medium and the surrounding flow path can introduce significant spatial variations in the local flow field. As a result, the local filtration velocity and mass flux may differ from the corresponding nominal or area-averaged values. Such spatial variations are relevant to the aerodynamic characterization of the sampling stage because they define the local transport conditions through the collection medium.

For the computational fluid dynamics (CFD) analysis, the microscopic structure of the filter was not explicitly resolved at the fibre scale. A direct representation of the individual fibres would require a computational mesh with spatial resolution several orders of magnitude smaller than the characteristic dimensions of the complete sampling system, resulting in a prohibitive computational cost. Instead, the filter medium was represented as a homogenized porous domain with equivalent macroscopic hydraulic resistance. This approach enables the overall pressure-loss characteristics of the filter to be incorporated into the system-level CFD model without explicitly resolving the internal fibre structure.

The porous-medium formulation was based on the Darcy–Forchheimer resistance model, in which the pressure gradient through the filter is represented as the combined contribution of a viscous resistance term and an inertial resistance term. The corresponding resistance coefficients characterize the macroscopic hydraulic response of the filter and provide an effective representation of the pressure loss associated with airflow through the porous medium.

The computational domain encompasses the principal regions of the air-sampling path, including the upstream flow region, the pleated H13 HEPA filter represented as a porous medium, and the downstream suction region. The CFD model therefore describes the single-phase aerodynamic behaviour of the sampling subsystem and is intended to characterize the pressure field, velocity field, and spatial distribution of mass flux throughout the filter assembly.

The present model does not explicitly resolve the individual filter fibres or the trajectories of discrete aerosol particles. Consequently, the simulations characterize the continuous-phase airflow and macroscopic hydraulic response of the filter, rather than particle-size-dependent filtration mechanisms, particle deposition, or aerosol collection efficiency. These phenomena require additional particle-transport and deposition modelling and are therefore outside the scope of the present aerodynamic analysis.

### 2.3. Three-Dimensional CFD Model

Following the modelling strategy described above, a three-dimensional (3D) CFD model was developed as the primary system-level representation of the MARE sampling configuration. The simulations were performed using STAR-CCM+ v14.06.012-R8 [16]. The 3D model was constructed to characterize the aerodynamic behaviour of the MARE system, with particular emphasis on the airflow distribution through the pleated H13 HEPA aerosol collection filter and the downstream suction assembly.

The three-dimensional formulation enables the spatial distribution of the flow field to be evaluated throughout the sampling configuration, including both circumferential and vertical variations in airflow. In particular, the model accounts for the coupling between the three-dimensional pleated-filter geometry and the downstream suction configuration, allowing the resulting pressure and velocity fields to be resolved at the system scale.

A complementary two-dimensional (2D) model was subsequently developed to investigate the local flow behaviour within a representative filter pleat. Unlike the 3D model, which describes the overall aerodynamic response of the sampling system, the 2D model was intended to isolate the local redistribution of airflow across the characteristic regions of an individual pleat. This reduced-order representation was therefore used to quantify variations in mass flux at the pleat scale. The two models consequently address different spatial scales: the 3D model provides the system-level aerodynamic characterization, whereas the 2D model provides a localized analysis of the flow distribution within a representative pleat.

To reduce the computational requirements while retaining the dominant flow physics, the geometric symmetry of the MARE configuration was exploited. Consequently, only one half of the complete three-dimensional geometry was included in the computational domain. A symmetry boundary condition was imposed on the corresponding symmetry plane, thereby reproducing the flow behaviour of the full configuration while reducing the number of computational cells required.

The computational domain was divided into three main regions:

- External fluid region, representing the ambient air volume surrounding the pleated H13 HEPA aerosol collection filter;
- Porous region, representing the pleated H13 HEPA aerosol collection filter modelled as a porous medium;
- Internal fluid region, corresponding to the volume downstream of the filter, including the suction duct and extraction pathway.

The computational geometry was established to provide a macroscopic representation of the principal airflow path through the MARE sampling system rather than to reproduce every structural feature of the physical instrument. Geometrical details that do not significantly influence the global aerodynamic response were therefore omitted. This approach allowed the dominant flow mechanisms, pressure losses, and spatial velocity and mass-flux distributions to be captured while maintaining a computationally tractable model.

The resulting flow path can be summarized as:

ambient air → upstream external fluid region → pleated H13 HEPA filter represented as a porous medium → downstream internal fluid region → suction duct → extraction outlet.

**Figure 2:**
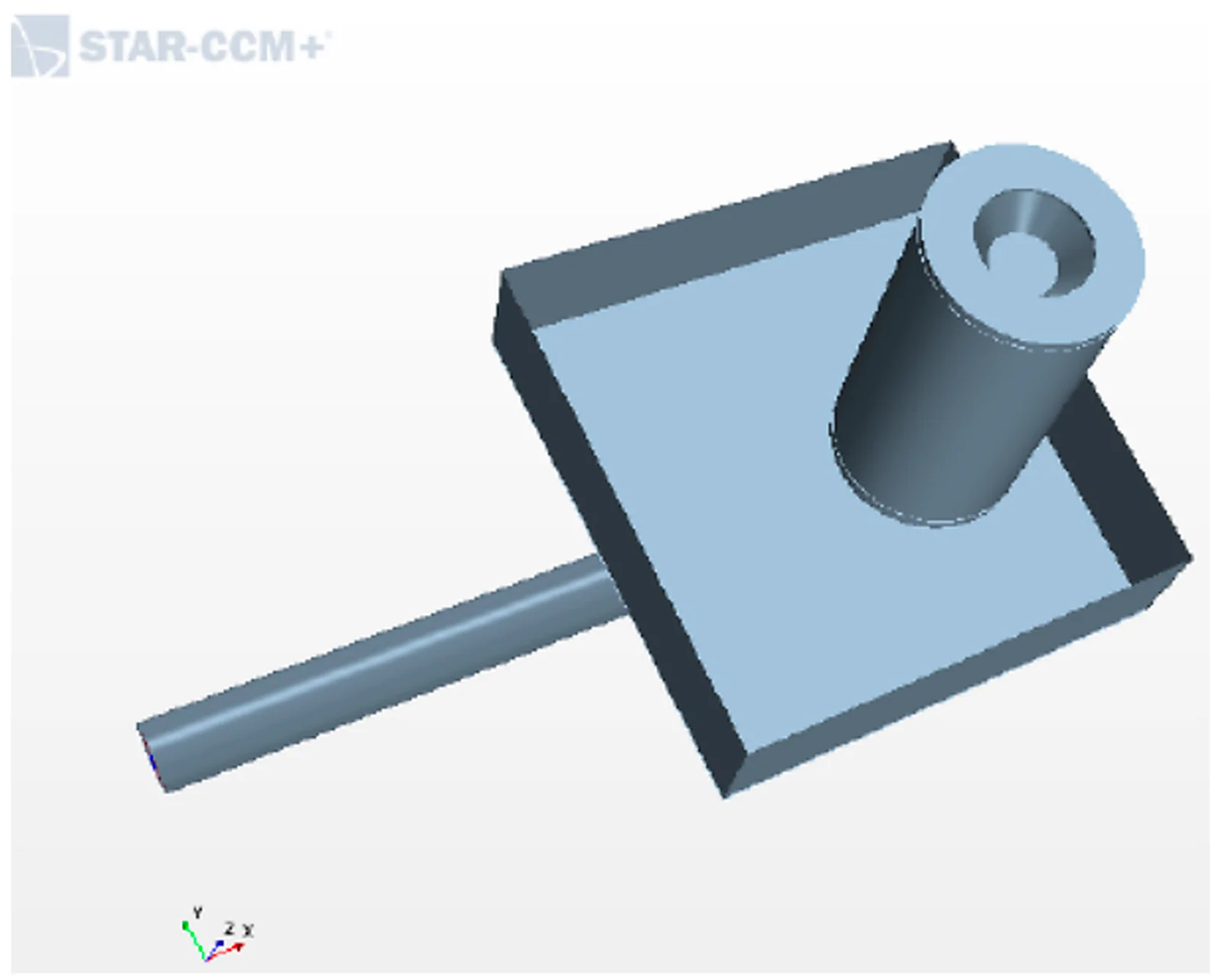
Three-dimensional CFD model of the MARE sampling system. The computational domain comprises the upstream external fluid region, the homogeneous porous region representing the pleated H13 HEPA aerosol collection filter, and the downstream internal fluid region leading to the suction pathway. The model exploits the geometric symmetry of the system and represents the nominal operating condition of 200 m^3^ h^−1^. Solid surfaces are treated as no-slip walls, while the symmetry plane is defined using a symmetry boundary condition.

This modelling approach enables the global pressure-drop behaviour of the sampling system and the spatial distribution of airflow through the pleated filter to be characterized simultaneously. The resulting aerodynamic information provides the basis for subsequent analyses of aerosol transport and collection, as well as for assessing the implications of non-uniform airflow on the downstream radiation-detection process. A representative view of the computational model is presented in Figure 2.

### 2.4. Computational Mesh

The three-dimensional computational domain was discretized using an unstructured polyhedral-cell mesh. Local mesh refinement was applied in regions where increased spatial resolution was required to adequately capture the flow field, particularly within and around the pleated H13 HEPA aerosol collection filter, along the downstream suction pathway, and at the main geometrical transitions. Prism layers were introduced adjacent to solid surfaces to improve the resolution of the near-wall flow region and the representation of wall-bounded velocity gradients.

The near-wall treatment was implemented using the two-layer all-(y+) wall treatment available in STAR-CCM+, consistently with the realizable (*k-ε*) turbulence model described in Section 2.5. This approach provides a suitable treatment of the near-wall region while maintaining compatibility with the turbulence modelling strategy adopted for the system-level simulations.

The final three-dimensional mesh comprised 834,815 polyhedral cells, including 95,909 cells within the porous filter region. The remaining cells were distributed between the upstream external-fluid region and the downstream internal-fluid region. The resulting distribution of computational cells is summarized in Table 1.

The pleated H13 HEPA filter region was explicitly refined because it represents the principal hydraulic resistance of the sampling system and constitutes the main region in which the airflow undergoes a significant change in flow area and associated pressure loss. Additional local refinement was introduced in the downstream suction region, where the airflow is redirected towards the extraction outlet and relatively strong spatial gradients in velocity and pressure may develop. Refinement was also applied at relevant geometrical transitions to reduce the numerical diffusion associated with abrupt changes in the flow path.

A formal mesh-independence or grid-convergence study involving multiple mesh resolutions was not performed in the present work. Consequently, the sensitivity of the predicted pressure drop and, in particular, the local mass-flux distribution to mesh resolution could not be quantitatively assessed. The selected mesh should therefore not be interpreted as providing a formally grid-independent solution.

In addition, characteristic minimum and maximum cell dimensions and detailed quantitative mesh-quality indicators were not systematically recorded for the original simulations. These parameters are therefore not reported as quantitative measures of mesh sensitivity. The numerical results should consequently be interpreted within the context of the adopted computational discretization.

Particular consideration is required when interpreting local mass-flux values, since spatially resolved quantities may exhibit greater sensitivity to mesh resolution than integrated or system-level quantities, such as the overall pressure drop. Accordingly, the analysis emphasizes systematic spatial distributions and flow trends rather than isolated local extrema. The adopted mesh was considered sufficient for the system-level aerodynamic characterization and for identifying the principal spatial variations in airflow through the pleated filter.

### 2.5. Physical Models and Air Properties

The airflow through the MARE sampling system was modelled as a steady-state, three-dimensional, incompressible flow with constant thermophysical properties. Under the operating conditions considered, variations in air density due to pressure and temperature changes were assumed to be negligible. Air was therefore treated as a constant-density Newtonian fluid, with constant density and dynamic viscosity throughout the computational domain.

The porous-medium parameters used to represent the pleated H13 HEPA aerosol collection filter were not obtained from an independent experimental characterization performed under the present CFD configuration. Instead, the Darcy–Forchheimer resistance coefficients were parameterized to reproduce the expected macroscopic pressure-drop behaviour of the filter at the nominal operating flow rate. This procedure provides a calibrated representation of the hydraulic resistance of the porous medium within the system-level CFD model. It should therefore be regarded as a model parameterization procedure rather than an independent experimental validation of the numerical model.

The following physical and numerical models were employed in the simulations:

- Three-dimensional flow;
- Steady-state formulation;
- Segregated flow solver;
- Constant-density gas model;
- Realizable k-ε turbulence model;
- Two-layer wall treatment;
- Second-order convection discretization scheme;
- Gravity effects;
- Hybrid Gauss–Least-Squares (Hybrid-Gauss-LSQ) gradient reconstruction;
- Venkatakrishnan limiter.

The thermophysical properties of air were assumed constant throughout the computational domain and were defined by the following values:

*ρ* = 1.18415 kg m^−3^

and

*μ* = 1.88508 × 10^−5^ Pa s

where *ρ* is the air density and *μ* is the dynamic viscosity.

The realizable *k-**ε*** turbulence model was selected to provide a robust and computationally efficient description of the turbulent flow field within the MARE sampling configuration. This formulation was considered appropriate for the complex internal flow geometry, including the contraction and acceleration of the airflow through the pleated H13 HEPA filter and the subsequent redirection of the flow towards the suction duct.

Near-wall effects were accounted for using the two-layer wall treatment available in STAR-CCM+. This treatment was applied consistently with the selected realizable (k)-(\varepsilon) turbulence formulation. The governing equations were discretized using a second-order convection scheme to improve the spatial accuracy of the predicted pressure and velocity fields while limiting numerical diffusion.

The Hybrid Gauss–Least-Squares gradient reconstruction was employed for the evaluation of spatial gradients, while the Venkatakrishnan limiter was used to control numerical oscillations in regions exhibiting strong flow gradients. Gravity was included in the simulations to account for its potential contribution to the pressure field, although its influence on the predominantly forced airflow was expected to be secondary under the operating conditions considered.

The combination of the above physical models and numerical schemes was selected to provide a computationally efficient representation of the system-level aerodynamic behaviour while retaining sufficient spatial resolution to characterize the pressure distribution, velocity field, and mass-flux variations through the pleated filter.

All modelling assumptions, material properties, boundary conditions, solver settings, and numerical discretization parameters used in the simulations were defined consistently throughout the CFD study and are documented in the corresponding simulation setup.

### 2.6. Porous-Medium Model

The pleated H13 HEPA aerosol collection filter was represented in the CFD model as a homogeneous porous medium, rather than by explicitly resolving its microscopic fibre structure. This homogenized representation allows the macroscopic hydraulic resistance of the filter to be incorporated into the system-level model while avoiding the prohibitive computational cost associated with fibre-scale resolution.

The pressure drop through the porous region was described using the Darcy–Forchheimer formulation [17]:

$$\frac{\Delta p}{L}=-\left(P_{i}|v|+P_{v}\right)v$$

where *P_i_* is the inertial resistance coefficient, *P_v_* is the viscous resistance coefficient and *v* is the superficial velocity through the porous medium.

The porous-medium parameters used in the present CFD model were derived from the experimentally based pressure-drop/permeability characterization reported by Innocentini et al. [18]. The parameters reported in that study were converted into the resistance-coefficient formulation required by the present CFD implementation. The conversion accounted for the definitions of superficial velocity, porous-medium thickness, fluid viscosity, and fluid density adopted in the respective formulations.

The parameters obtained from the characterization reported by Innocentini et al. [18] were:

*k*_1_ = 1.34 × 10^−12^ m^2^

and

*k*_2_ = 2.71 × 10^−7^ m.

Following their conversion to the resistance-coefficient formulation used in STAR-CCM+, the corresponding porous-medium coefficients were:

*P_i_* = 3.524256 × 10^4^ kg · m^−4^

and

*P_v_* = 2.72998 × 10^−4^ Pa · s · m^−2^.

The parameters reported by Innocentini et al. [18] were obtained from silicon-carbide foam filters, rather than from the specific H13 HEPA filter installed in the MARE system. Consequently, the resulting coefficients should not be interpreted as intrinsic or independently measured hydraulic properties of the MARE filter. Instead, they were adopted as effective macroscopic resistance parameters for representing the filter as a homogeneous porous region within the system-level CFD model.

Using the resulting Darcy–Forchheimer coefficients, the calculated pressure drop across the filter at the nominal operating flow rate of 200 m^3^ h^−1^ was 268.697 Pa. This value was compared with the initial pressure-drop range of approximately 250–300 Pa reported for H13 HEPA filters in the technical documentation provided by SITASA, Suministros Industriales del Tajo (AFF Internacional, 2001) [13], and in the AFPRO FILTERS Product Catalogue (2019) [14]. The calculated pressure drop lies within this reference range, indicating consistency between the macroscopic hydraulic response predicted by the CFD model and the pressure-drop values reported for comparable H13 HEPA filter configurations.

This comparison is considered a consistency check rather than an independent experimental validation of the CFD model. The reference pressure-drop values in [13,14] correspond to technical documentation for H13 HEPA filters and do not represent direct measurements of the complete MARE sampling subsystem under the same experimental and operating conditions. Furthermore, the agreement with the 250–300 Pa range was not used to modify or recalibrate the resistance coefficients derived from Innocentini et al. [18]. Instead, the comparison was used solely to assess whether the resulting effective porous-medium representation produced a pressure-loss magnitude consistent with the documented hydraulic behaviour of comparable H13 HEPA filters.

The adopted Darcy–Forchheimer coefficients therefore represent the effective viscous and inertial contributions to the pressure loss through the homogenized porous region. Their numerical values are dependent on the specific porous-medium formulation, the definitions of superficial velocity and material properties, the filter thickness, and the units and conventions adopted by the CFD solver. Accordingly, the coefficients should be interpreted within the framework of the present CFD implementation rather than as universal material properties of the H13 filter medium.

The adopted porous-medium formulation enables the macroscopic hydraulic resistance of the pleated filter to be incorporated into the complete MARE system model while avoiding explicit resolution of the microscopic fibre structure. This approach provides a computationally tractable representation of the filter pressure loss and allows the resulting system-level pressure and airflow distributions to be evaluated under the nominal operating conditions.

### 2.7. Boundary Conditions

The suction effect generated by the pump was represented by imposing the extraction condition at the downstream end of the sampling pathway. The downstream section was defined as a velocity inlet boundary with a prescribed velocity directed into the computational domain, corresponding to the suction flow generated by the pump. The nominal operating condition considered in the simulations was a volumetric flow rate of 200 m^3^ h^−1^.

The external spherical domain surrounding the sampling assembly was defined using a pressure outlet boundary condition, representing the connection of the computational domain to ambient atmospheric air. The reference pressure at this boundary was set to atmospheric pressure.

The pleated H13 HEPA filter was assigned the previously defined homogeneous porous-medium formulation, including the corresponding Darcy and Forchheimer resistance coefficients. The symmetry plane resulting from the geometric reduction of the computational domain was defined using a symmetry boundary condition. All solid surfaces, including the filter housing, detector supports, covers, and internal piping, were treated as no-slip walls, imposing zero fluid velocity relative to the solid surfaces.

The Darcy–Forchheimer resistance coefficients used to represent the filter were selected to provide an effective macroscopic hydraulic resistance consistent with the expected pressure-drop behaviour of the H13 HEPA filter. The parameterization was based on the initial pressure-drop range of approximately 250–300 Pa reported for the filter under the relevant operating conditions. The selected porous-medium parameters resulted in a pressure drop within this reference range at the nominal flow rate of 200 m^3^ h^−1^.

The agreement between the numerically predicted pressure drop and the reported 250–300 Pa range is interpreted as a calibration and consistency assessment of the porous-medium representation, rather than as an independent experimental validation of the complete CFD model. The reference pressure-drop values do not correspond to direct measurements of the complete MARE sampling subsystem under identical experimental conditions. Therefore, the boundary conditions and porous-medium parameters were selected to provide a physically consistent representation of the nominal operating condition, while the resulting CFD predictions are interpreted within the limitations of the adopted modelling assumptions.

All simulations presented in this study were conducted at the nominal volumetric flow rate of 200 m^3^ h^−1^, corresponding to the specified operating condition of the MARE sampling subsystem.

### 2.8. Two-Dimensional Filter Model

Two complementary numerical models were developed to characterize the aerodynamic behaviour of the pleated H13 HEPA aerosol collection filter in the MARE system. A three-dimensional (3D) model was first established to represent the complete filter geometry and to characterize the spatial distribution of the airflow throughout the full sampling configuration. A simplified two-dimensional (2D) model was subsequently developed based on a representative filter pleat to investigate the local flow behaviour and pressure drop at the pleat scale while substantially reducing the computational cost.

The two modelling approaches were not intended to constitute independent validation cases. Rather, the 2D model was employed as a complementary reduced-order representation of the filter to assess whether the principal hydraulic behaviour identified in the 3D model was preserved when the geometry and computational domain were simplified.

In particular, the pressure-drop predictions obtained from the 3D and 2D models were compared as a model-to-model consistency check. This comparison was used to evaluate the degree to which the reduced 2D representation reproduced the macroscopic hydraulic response of the filter predicted by the full 3D configuration. Differences between the two predictions were interpreted in the context of the different geometric representations and spatial scales addressed by each model, rather than as evidence of numerical or experimental validation.

The 3D model therefore provides the system-level aerodynamic characterization, including the circumferential and vertical distribution of airflow through the complete pleated filter, whereas the 2D model provides a localized pleat-scale characterization of the flow field and pressure loss. Together, the two approaches provide complementary information on the hydraulic behaviour of the pleated filter while maintaining a computationally tractable modelling strategy.

#### 2.8.1. Numerical Convergence

The simulations were performed using a steady-state formulation. The iterative solution was continued until the monitored flow variables exhibited stable behaviour and no significant changes in the global flow field were observed between successive iterations. Particular attention was given to the pressure drop across the porous filter region and to the mass-flux distribution at the filter outlet, which were monitored throughout the iterative solution process.

The numerical solution was considered sufficiently converged for the purposes of the present hydraulic characterization when the monitored quantities reached a stable behaviour. This convergence criterion was applied consistently to both the three-dimensional (3D) and two-dimensional (2D) simulations.

However, a formal residual-based convergence assessment was not performed. In particular, different residual convergence thresholds were not systematically investigated, nor was the asymptotic variation in the monitored engineering quantities quantified through a dedicated convergence study. Consequently, no formal numerical convergence uncertainty can be assigned to the reported pressure-drop and local mass-flux values.

This limitation is particularly relevant to the interpretation of local mass-flux distributions, since spatially resolved quantities may exhibit greater sensitivity to numerical discretization and local velocity gradients than integrated quantities such as total flow rate or overall pressure drop. Therefore, local mass-flux results are interpreted primarily in terms of their spatial distribution, relative variations, and systematic differences between characteristic regions of the filter, rather than as exact pointwise predictions.

#### 2.8.2. Three-Dimensional Model

The 3D model was developed to represent the complete pleated-filter geometry and the surrounding upstream and downstream flow regions. The geometric symmetry of the MARE configuration was exploited, and consequently only one half of the complete filter assembly was included in the computational domain. A symmetry boundary condition was imposed on the corresponding symmetry plane.

The computational domain comprised three principal regions: the upstream external-fluid region, the pleated H13 HEPA aerosol collection filter represented as a porous medium, and the downstream internal-fluid region leading to the suction system.

The filter was modelled as a homogeneous porous medium using the Darcy–Forchheimer formulation described previously. This approach represents the macroscopic hydraulic resistance of the filter without explicitly resolving its microscopic fibre and pore structure. The corresponding porous-medium coefficients were selected according to the parameterization procedure described in Section 2.6.

The computational mesh was generated using polyhedral cells, with prism layers applied adjacent to solid surfaces to improve the representation of the near-wall flow. The final 3D mesh contained 834,815 cells, distributed as follows: 625,227 cells in the upstream external-fluid region, 95,909 cells in the porous filter region, and 113,679 cells in the downstream internal-fluid region.

Air was modelled as an incompressible fluid with constant density and constant thermophysical properties. Turbulence was described using the realizable (k)-(\varepsilon) model with a two-layer wall treatment. The governing equations were discretized using second-order convection schemes. Spatial gradients were evaluated using the Hybrid Gauss–Least-Squares method, and the Venkatakrishnan limiter was employed to improve the numerical treatment of regions exhibiting strong flow gradients.

The suction effect of the downstream pumping system was represented by a prescribed velocity boundary condition at the downstream section of the computational domain. The imposed velocity was determined from the nominal volumetric flow rate of 200 m^3^ h^−1^. The external spherical boundary was defined as a pressure outlet representing the connection with ambient atmospheric air. All solid surfaces, including the housing, detector supports, covers, and piping, were treated as no-slip walls. Gravity was included in the simulations.

The pressure drop across the porous filter was calculated from the difference between representative pressure levels upstream and downstream of the filter. At the nominal operating flow rate of 200 m^3^ h^−1^, the 3D model predicted a filter pressure drop of 268.697 Pa.

The spatial distribution of airflow through the filter was characterized using 21 horizontal sections and 31 vertical sections. The horizontal sections were used to evaluate circumferential variations in mass flux, whereas the vertical sections were used to characterize variations along the filter height. The results indicated a comparatively uniform circumferential distribution, while more pronounced variations were observed in the vertical direction. In particular, higher mass-flux values were obtained in the lower portion of the filter, in the vicinity of the downstream suction region.

#### 2.8.3. Two-Dimensional Representative Model

A simplified 2D model was developed as a complementary representation of the hydraulic behaviour of a representative filter pleat. The geometry was derived from the characteristic dimensions of the 3D filter and comprised the upstream external-fluid region, the pleated H13 HEPA filter material, and the downstream internal-fluid region.

The 2D representation retained the principal geometrical features governing the flow through an individual pleat while substantially reducing the computational domain and associated computational cost. As in the 3D model, the filter material was represented as a homogeneous porous medium using the same Darcy–Forchheimer resistance formulation.

The inlet and outlet conditions were defined to reproduce an operating condition consistent with the 3D simulation. In particular, the characteristic mean velocity imposed in the 2D model was derived from the mean outlet velocity obtained from the 3D calculation. This approach enabled the local hydraulic response of a representative pleat to be examined under conditions consistent with the system-level solution.

The mean mass flux obtained from the 3D model was 1.36444 kg m^−2^ s^−1^. The representative 2D model reproduced the same general spatial trend observed in the 3D analysis, with higher mass-flux values in the internal region of the pleat and lower values in the external region. Relative to the mean mass flux, the internal region exhibited an increase of approximately 30%, whereas the external region exhibited a reduction of approximately 10%.

The pressure drop predicted by the representative 2D model was 262.3108 Pa, compared with 268.697 Pa obtained from the 3D model. The absolute difference was therefore 6.39 Pa, corresponding to approximately 2.4% of the 3D pressure-drop value.

The comparison between the two models is interpreted as a model-to-model consistency assessment rather than an independent validation. The 2D model is not an independent experimental or numerical reference because its operating condition was derived from the 3D solution and its geometric representation differs substantially from the complete three-dimensional configuration. The comparison therefore assesses whether the principal hydraulic response of the filter is preserved when the system is reduced to a representative pleat-scale geometry.

The close agreement between the predicted pressure drops indicates consistency between the two modelling approaches at the global hydraulic level. The 3D model was nevertheless retained as the primary basis for analysing the spatial distribution of mass flux throughout the complete pleated filter, whereas the 2D model was used to investigate the local flow redistribution within a representative pleat.

### 2.9. Mass-Flux Analysis

Mass flux was selected as the principal variable for characterizing the spatial distribution of airflow through the pleated filter. Unlike the global pressure drop, which provides an integrated measure of the hydraulic resistance of the filter, the mass-flux distribution provides spatially resolved information on the local airflow reaching different portions of the collection surface.

For the 3D model, the filter was analysed using 21 horizontal sections and 31 vertical sections. The horizontal sections were used to characterize the circumferential distribution of mass flux, whereas the vertical sections were used to evaluate variations in mass flux along the height of the filter. This analysis enabled systematic spatial trends to be identified without relying exclusively on isolated local extrema.

For the representative 2D model, the mass-flux distribution was evaluated using line probes positioned along the representative pleat. The resulting profiles were used to characterize the redistribution of airflow between the characteristic internal and external regions of the pleat.

The simplified 2D geometry contains sharp vertices that do not reproduce the rounded geometry of the actual pleated filter. These geometrical discontinuities produced localized numerical artefacts and non-physical mass-flux values in the immediate vicinity of the vertices. The affected probe points were therefore excluded from the quantitative analysis of the overall mass-flux distribution.

Accordingly, the mass-flux results presented in this work are interpreted primarily in terms of regional distributions, relative deviations from the mean value, and systematic spatial trends. This approach minimizes the influence of localized numerical artefacts and is consistent with the absence of a formal mesh-independence and numerical-convergence uncertainty assessment.

## 3. Results

### 3.1. Pressure Drop

The pressure drop across the pleated H13 HEPA aerosol collection filter was evaluated for both the complete three-dimensional (3D) model and the representative two-dimensional (2D) model. The nominal operating condition considered in both cases corresponded to a volumetric flow rate of 200 m^3^ h^−1^.

For the complete 3D model, the calculated pressure drop across the porous filter region was:

Δ*P*_3*D*_ = 268.7 Pa

As a technical reference for the macroscopic hydraulic behaviour of the filter, an initial pressure-drop range of approximately 250–300 Pa was considered. This range was obtained from technical documentation provided by SITASA, Suministros Industriales del Tajo (AFF Internacional, 2001) [13], and the AFPRO FILTERS Product Catalogue (2019) [14], which report initial pressure-drop values in this range for H13 HEPA filters under relevant operating conditions.

The predicted pressure drop of 268.697 Pa therefore lies within the adopted technical reference range. This agreement is interpreted as a consistency and parameterization assessment of the effective porous-medium representation. It should not be regarded as an independent experimental validation of the CFD model, since the reference values reported in [13,14] do not correspond to direct measurements of the complete MARE sampling subsystem under identical operating and experimental conditions.

The representative 2D model yielded a pressure drop of:

Δ*P*_2*D*_ = 262.31 Pa

The absolute difference between the two numerical predictions was calculated as:

Δ*P*_3*D*_ − Δ*P*_2*D*_ = 6.39 Pa,

corresponding to approximately 2.4%.

The comparison between the two models is summarized in Table 2.

The relatively small difference between the two predictions indicates a consistent macroscopic hydraulic response between the complete 3D representation and the simplified representative-pleat model. However, this comparison constitutes a model-to-model consistency check rather than an independent validation or uncertainty estimate, because the operating condition of the 2D model was derived from the 3D solution and the two models differ in dimensionality, geometry, and boundary-condition representation.

Accordingly, the 3D pressure-drop prediction is considered the primary system-level result, while the 2D calculation provides complementary evidence that the principal hydraulic response of the porous filter is preserved when the geometry is reduced to a representative pleat. The comparison therefore supports the use of the 2D model for subsequent local analysis of the pleat-scale mass-flux distribution.

### 3.2. Pressure and Velocity Fields

The CFD results show that sampled air enters the external fluid region surrounding the pleated H13 HEPA aerosol collection filter and is subsequently drawn through the porous filter medium toward the downstream suction duct. The resulting flow field is driven by the pressure gradient imposed by the extraction system, which establishes the overall transport of air from the ambient environment, through the filtration stage, and into the downstream suction pathway.

The streamline distribution indicates that the airflow is strongly influenced by the internal geometry of the sampling system. Local acceleration and deceleration occur near structural components, geometric transitions, and regions where the flow is redirected toward the suction duct. These effects produce spatial variations in velocity throughout the computational domain and result in a non-uniform approach flow at the filter surface.

The computed pressure field indicates that the principal pressure loss occurs across the pleated H13 HEPA filter, which constitutes the dominant hydraulic resistance of the sampling system. Additional pressure variations are observed downstream of the filter and are associated with flow contraction, redirection, and transport through the suction pathway. These features reflect the redistribution of the flow as it passes from the filtration region into the extraction system.

Overall, the results demonstrate that the flow approaching the filter is non-uniform and is significantly affected by the complete geometry of the air-sampling configuration. Consequently, the local velocity and mass-flux distributions at the filter surface may influence the subsequent transport and collection of aerosol particles. These findings emphasize the importance of considering the complete sampling-system geometry when characterizing the aerodynamic behaviour of the filter and assessing the spatial distribution of airflow through the filtration stage.

### 3.3. Circumferential Distribution of Mass Flux

The analysis of the 21 horizontal cross-sections indicates that, at a given elevation, the mass-flux distribution along the filter circumference remains relatively uniform. The variations observed within individual cross-sections are comparatively small, indicating a limited dependence of the local mass flux on the circumferential position around the filter.

These results indicate that the dominant spatial variation in mass flux occurs in the vertical direction rather than along the circumferential coordinate. Accordingly, the airflow distribution is primarily governed by height-dependent effects associated with the interaction between the pleated filter geometry and the downstream suction configuration.

The relatively uniform circumferential distribution indicates that the suction arrangement does not produce pronounced azimuthal asymmetries at equivalent elevations. No preferential concentration of airflow toward specific circumferential regions is observed, resulting in a comparatively balanced distribution of mass flux around the filter perimeter at a given height.

However, the circumferential uniformity should not be interpreted as evidence of a spatially homogeneous flow field over the entire filter surface. The CFD results show substantial variations in mass flux with elevation, leading to pronounced vertical gradients in the local filtration velocity. Consequently, different regions of the filter are subjected to different local airflow loads, which may affect the spatial distribution of aerosol transport and deposition. Such effects, however, cannot be quantitatively resolved with the present continuous-phase CFD model, which does not explicitly simulate discrete aerosol-particle trajectories or filtration mechanisms at the fibre scale.

Overall, the results indicate that the airflow exhibits a predominantly uniform circumferential distribution at a given elevation, whereas the overall spatial distribution of mass flux is primarily controlled by vertical variations induced by the geometry of the sampling and suction system. This behaviour supports the use of vertical cross-sectional analysis as the principal approach for characterizing the spatial non-uniformity of airflow through the pleated filter.

### 3.4. Vertical Distribution of Mass Flux

The vertical analysis of the mass-flux distribution reveals a pronounced spatial non-uniformity over the filter surface. The CFD results indicate that the highest mass-flux values occur in the lower portion of the pleated H13 HEPA filter, where the local filtration velocity is consequently greater than in the central region. Within the present model, this behaviour is primarily associated with the proximity of the lower filter section to the suction duct, where the extraction system exerts a stronger influence on the local flow field.

A secondary increase in mass flux is observed near the upper boundary of the filter. Although this increase is less pronounced than that identified in the lower region, it indicates that the flow distribution is also influenced by geometric and boundary effects at the upper extremity of the sampling configuration.

**Figure 3:**
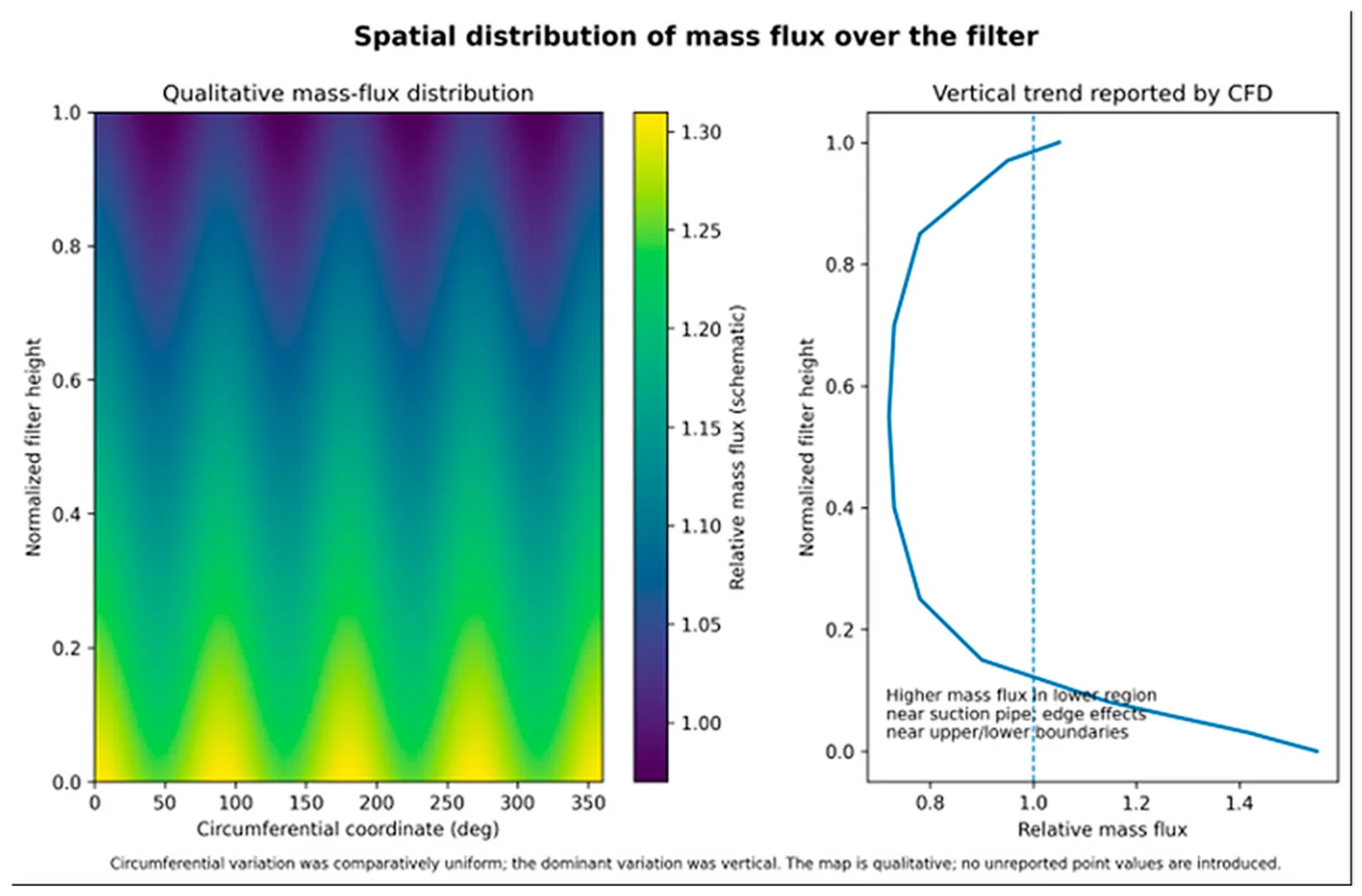
Spatial distribution of mass flux (kg m^−2^ s^−1^) over the pleated H13 HEPA aerosol collection filter obtained from the three-dimensional CFD model at a volumetric flow rate of 200 m^3^ h^−1^. The colour scale represents the local mass flux through the filter surface. The distribution is comparatively uniform in the circumferential direction, whereas the dominant spatial variation occurs along the vertical direction, with increased mass flux in the lower region near the suction pathway.

Based on the vertical distribution, three characteristic regions can be identified (Figure 3):

- Relatively high mass flux values near the lower boundary;
- Lower and more uniform mass flux values throughout the central region;
- A secondary increase in mass flux close to the upper boundary.

These results demonstrate that the local airflow through the filter is strongly dependent on vertical position. In particular, the enhanced mass flux in the lower region indicates that equivalent filter surface areas are subjected to different local filtration velocities. Therefore, the nominally available filter area is not hydraulically utilised under spatially uniform conditions, and the local airflow loading varies substantially along the filter height.

The predicted aerodynamic non-uniformity may influence the subsequent transport and deposition of aerosol particles by modifying the local flow conditions at the filter surface. However, the present continuous-phase CFD model does not resolve discrete particle trajectories, particle deposition, or the spatial accumulation of radioactive material. Consequently, the present results cannot be used to establish whether the predicted mass-flux variations lead to corresponding spatial differences in aerosol deposition or collected radioactive activity. Quantification of these effects would require dedicated particle-transport and deposition modelling and/or experimental measurements.

From an aerosol-sampling perspective, these results highlight the importance of characterising the local airflow distribution rather than relying exclusively on the mean filtration velocity or total volumetric flow rate. The observed vertical non-uniformity demonstrates that the suction-system geometry plays a significant role in determining the spatial distribution of airflow through the filter and, consequently, the local aerodynamic loading of the available collection area.

### 3.5. Flow Distribution Within a Representative Pleat

The two-dimensional model provides a higher-resolution representation of the local flow distribution within a representative pleat of the H13 HEPA filter. The analysis enables the spatial variation in mass flux to be examined at the pleat scale, complementing the system-level results obtained from the three-dimensional model.

The mean mass flux obtained for the representative pleat was:

$$\bar{G}=1.36444\mathrm{kg}\;m^{-2}\;s^{-1}$$

The mass-flux distribution exhibits a clear spatial variation between the different regions of the pleat. The internal region, located closer to the downstream suction pathway, presents the highest local mass-flux values, reaching approximately 30% above the mean value. In contrast, the external region exhibits lower mass-flux values, approximately 10% below the mean.

These differences indicate that the local airflow through the pleat is not spatially uniform, even at the scale of a single representative filter element. The observed distribution is consistent with the influence of the downstream extraction configuration on the local pressure and velocity fields within the pleated geometry.

Consequently, the aerodynamic conditions within an individual pleat cannot be adequately characterised by a single uniform filtration velocity. Instead, the local mass flux varies according to the position within the pleat, reflecting the spatial redistribution of airflow induced by the filter geometry and the suction pathway. This result further supports the use of spatially resolved mass-flux distributions when characterising the aerodynamic behaviour of the pleated filter.

### 3.6. Local Numerical Effects

The detailed two-dimensional analysis revealed localized irregularities in the mass-flux distribution in the vicinity of the artificial vertices introduced by the simplified pleat geometry. These deviations were spatially confined to the immediate surroundings of the geometric discontinuities and differed from the broader mass-flux trends observed along the remainder of the filter surface.

These localized effects are attributed to the idealized geometric representation adopted in the two-dimensional CFD model. In the physical filter, the pleat tips and transitions between adjacent surfaces are rounded and continuous, whereas the simplified computational geometry contains sharp corners and vertices to facilitate mesh generation and reduce geometric complexity. Such idealizations can locally modify the velocity and pressure fields and generate artificially high or low mass-flux values in the immediate vicinity of the discontinuities.

Because these localized deviations are primarily associated with geometric idealization rather than with the resolved aerodynamic behaviour of the physical filter, the corresponding points were excluded from the quantitative assessment of the overall mass-flux distribution. The interpretation of the numerical results was therefore based on the spatially consistent trends observed away from the artificial vertices, rather than on isolated local extrema associated with the simplified geometry.

This treatment limits the influence of geometry-induced numerical artefacts on the interpretation of the CFD results and prevents localized numerical deviations from being interpreted as representative features of the actual filter. The resulting assessment therefore focuses on the resolved macroscopic flow behaviour and the principal spatial trends in mass flux within the representative pleat.

## 4. Discussion

### 4.1. Airflow as a Component of the Radiation Measurement Chain

A key implication of the present study is that the air-sampling process should be regarded as an integral component of the measurement chain in radioactive aerosol monitoring systems. In the MARE system, ambient air is actively transported through a pleated H13 HEPA aerosol collection filter, while a CeBr_3_ scintillation detector is positioned within the filter assembly to measure the radioactive material accumulated during sampling.

The overall measurement process can therefore be conceptualized as a sequence of interconnected stages: (i) air intake and transport through the sampling system, (ii) aerosol transport and collection by the filter, (iii) accumulation of radioactive material on the collection medium, and (iv) detection and quantification of the resulting radioactive emissions by the CeBr_3_ detector. The present study focuses primarily on the first stage and characterizes the aerodynamic conditions established upstream of the subsequent particle-collection and radiation-detection processes.

The relevance of this aerodynamic characterization arises from the spatially non-uniform nature of the sampling flow. The CFD results demonstrate that the local mass flux through the filter varies with position, with the most pronounced variations occurring along the vertical direction. Consequently, different regions of the collection surface are subjected to different local airflow conditions. These variations do not, by themselves, demonstrate corresponding spatial differences in aerosol deposition or radioactive activity; rather, they identify non-uniform aerodynamic conditions that may influence subsequent particle transport and collection.

This distinction is important when considering the complete radiation-monitoring chain. The present CFD model does not resolve discrete aerosol-particle trajectories, deposition mechanisms, radioactive activity accumulation, or the response of the CeBr_3_ detector. Instead, it provides a spatially resolved description of the airflow field that can serve as an input to subsequent particle-transport and deposition analyses. The resulting particle-deposition distribution could subsequently be coupled with radiation-transport and detector-response models to assess whether spatially non-uniform sampling conditions produce a measurable effect on the final radiological signal.

Within this framework, the air-sampling subsystem should not be considered independently from the radiation-detection stage. Rather, it constitutes the upstream aerodynamic component that establishes the flow conditions under which radioactive aerosol particles are transported to and collected by the filter, thereby defining the conditions preceding subsequent radiation detection.

The present results also demonstrate that global hydraulic quantities alone are insufficient to fully characterize the aerodynamic behaviour of the sampling system. Although the overall pressure drop provides an important measure of the hydraulic resistance of the filter assembly, it does not describe the spatial redistribution of airflow over the collection surface. The three-dimensional CFD model predicted a pressure drop of 268.697 Pa at a volumetric flow rate of 200 m^3^ h^−1^, while the representative two-dimensional model yielded 262.311 Pa under the corresponding operating condition. The resulting difference was approximately 2.4%.

Because the operating condition of the two-dimensional model was derived from the three-dimensional solution, this comparison should not be interpreted as an independent validation, accuracy assessment, or numerical uncertainty estimate. Instead, the close agreement provides a model-to-model consistency check, indicating that the simplified two-dimensional representation reproduces a comparable global hydraulic response while retaining its complementary role in resolving local flow redistribution within a representative filter pleat.

Overall, the combined results show that the aerodynamic behaviour of the sampling system constitutes an important upstream component of the radioactive aerosol measurement chain. The spatially resolved CFD analysis complements the global pressure-drop characterization by identifying local variations in airflow that would not be captured by integral hydraulic quantities alone. These results provide a physically consistent basis for subsequent investigations of aerosol transport, deposition, activity distribution, and their potential influence on the radiological measurement response.

### 4.2. Global Pressure Drop Does Not Fully Describe Flow Distribution

The pressure-drop predictions obtained from the three-dimensional and representative two-dimensional models are in close agreement, with a relative difference of approximately 2.4%. This agreement indicates that both modelling approaches reproduce a comparable global hydraulic response and overall flow resistance under the considered operating condition.

However, agreement in the integrated pressure drop does not imply a uniform spatial distribution of airflow through the filter. The three-dimensional results reveal significant variations in local mass flux over the filter surface, demonstrating that a single global hydraulic parameter cannot fully characterize the spatial structure of the flow field. This distinction is particularly relevant for radioactive aerosol monitoring systems, in which the local aerodynamic conditions established at the collection surface constitute the initial conditions for subsequent aerosol transport and collection.

The pressure drop across the filter can be expressed as the difference between the pressure levels upstream and downstream of the filtration medium:

$$\Delta P=f\left(\dot{Q},\mathrm{filter properties},\mathrm{flow conditions}\right)$$

Such a measurement does not provide information regarding the spatial distribution of flow within the filter:

***G*** = ***G***(***x***, ***y***, ***z***)

where ***G*** represents the local mass flux. Knowledge of this spatial distribution is essential for assessing whether all regions of the collection surface operate under comparable sampling conditions. Consequently, CFD modelling provides complementary information that cannot be obtained through conventional pressure-drop measurements alone.

**Figure 4:**
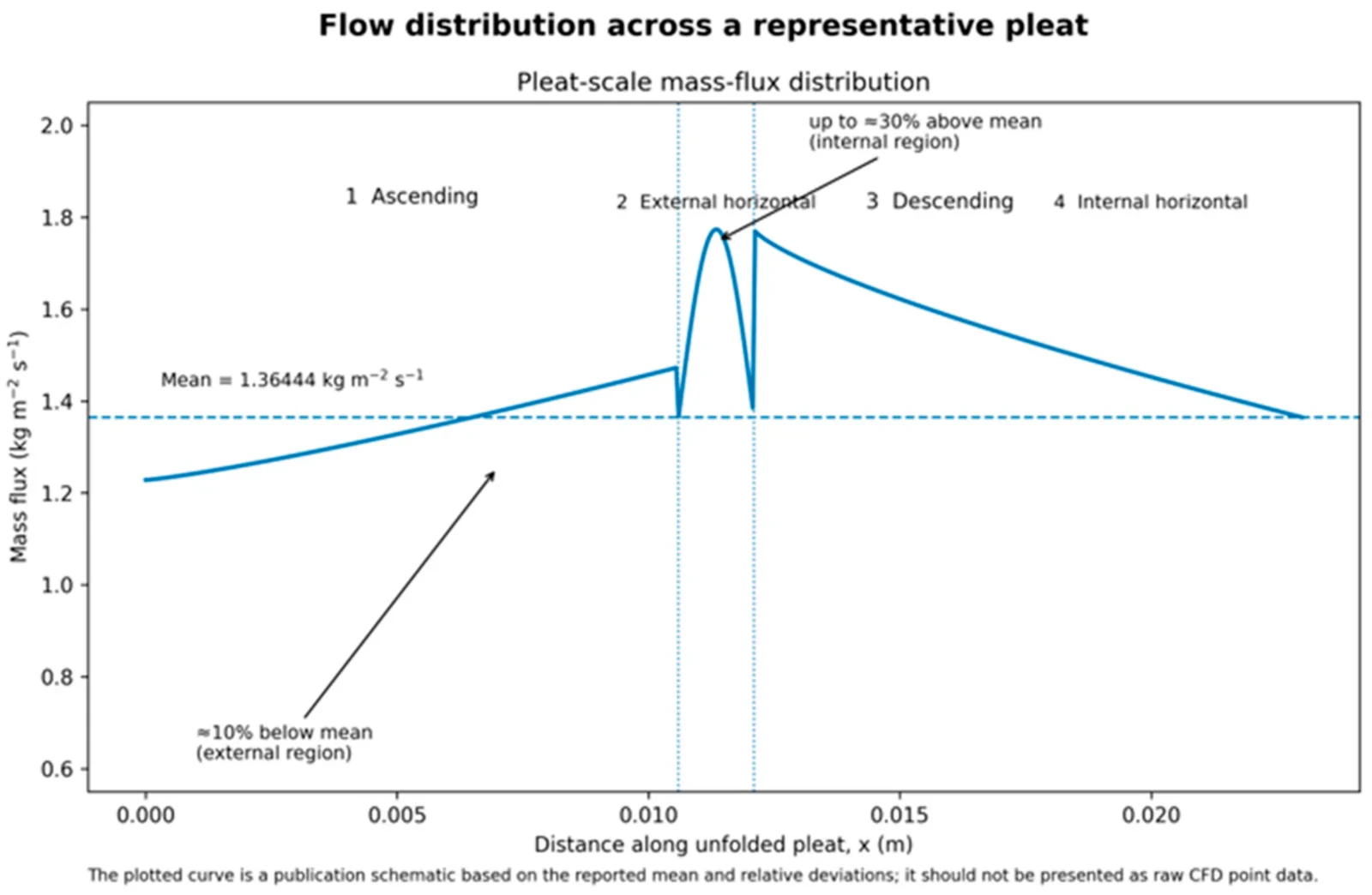
Local mass-flux distribution (kg m^−2^ s^−1^) obtained from the two-dimensional CFD solution along a representative pleat of the H13 HEPA filter. The colour scale represents the calculated local mass flux. The mean mass flux is 1.36444 kg m^−2^ s^−1^; the internal region exhibits values approximately 30% above the mean, whereas the external region exhibits values approximately 10% below the mean. Local points affected by the artificial sharp vertices of the simplified pleat geometry were excluded from the interpretation of the overall distribution.

The two-dimensional representative-pleat model further demonstrates the importance of this spatial resolution. The mass-flux distribution varies systematically across the different regions of the pleat. The internal region, located closer to the downstream suction pathway, exhibits mass-flux values approximately 30% above the mean, whereas the external region presents values approximately 10% below the mean. The resulting spatial distribution along the representative pleat is shown in Figure 4.

The observed mass-flux non-uniformity should not be interpreted as direct evidence of a corresponding non-uniformity in radioactive aerosol deposition or activity accumulation. The present CFD model resolves the continuous-phase airflow field but does not explicitly simulate discrete aerosol-particle transport, including particle inertia, Brownian diffusion, deposition mechanisms, or particle–surface interactions. Consequently, the approximately 30% higher mass flux predicted in the internal region identifies an area of increased local air transport through the filter, but does not provide a quantitative estimate of the aerosol mass or radioactive activity subsequently deposited in that region.

Nevertheless, the spatial variation in mass flux is relevant to the sampling process because it defines different local aerodynamic conditions for aerosol particles approaching and traversing the filter. The results therefore demonstrate that the global pressure drop should be complemented by spatially resolved flow-field information when characterizing the aerodynamic behaviour of the sampling filter. In this context, the CFD-derived mass-flux distribution provides a necessary aerodynamic basis for subsequent investigations of particle transport, deposition, and the potential influence of spatially non-uniform sampling conditions on radiation-measurement performance.

### 4.3. Implications for Radioactive Aerosol Collection

The CFD results indicate that the lower portion of the filter is subjected to higher local mass-flux values than the remaining collection surface. This indicates that, at a given operating condition, a greater mass flow rate of air passes through these regions per unit filter area and unit time. In addition, the two-dimensional representative-pleat analysis demonstrates that substantial spatial variations in mass flux may occur even within an individual pleat, with local values deviating from the corresponding mean mass flux.

From an aerosol-transport perspective, these spatial variations identify regions of the filter surface characterized by different local aerodynamic conditions. Such variations may modify the flow environment experienced by airborne particles as they approach and traverse the filtration medium. However, the present study does not establish a direct relationship between the predicted mass-flux distribution and particle trajectories, deposition rates, collection efficiency, or the spatial distribution of accumulated radioactive material.

These quantities depend on particle-specific properties and transport mechanisms, including particle inertia, Brownian diffusion, gravitational effects, and particle–surface interactions, which are not explicitly represented in the present continuous-phase CFD formulation. Consequently, the predicted mass-flux non-uniformities should be interpreted as aerodynamic indicators of spatially varying sampling conditions, rather than as direct measures of aerosol deposition or radioactive activity accumulation.

The present results therefore provide a spatially resolved aerodynamic basis for subsequent particle-transport and deposition analyses. Coupling the calculated flow field with an appropriate discrete-particle or aerosol-transport model would enable the potential relationship between local airflow conditions and aerosol collection to be investigated quantitatively.

### 4.4. Implications for Detector Measurements

The CeBr_3_ scintillation detector is positioned within the internal volume of the pleated filter and measures the gamma-ray emission associated with radioactive material accumulated during aerosol sampling. Consequently, the radiological measurement constitutes the final stage of a sequence of coupled aerodynamic and aerosol-collection processes that govern the transport and spatial accumulation of airborne radioactive material on the filter.

The CFD results presented in this study characterize the aerodynamic conditions preceding particle collection and radiation detection. The predicted spatial variations in mass flux identify regions of the filter subjected to different local airflow conditions. These variations should not be interpreted as direct indicators of spatial differences in radioactive activity, since discrete particle transport, deposition, and activity accumulation are not explicitly modelled in the present CFD framework. Nevertheless, the results provide a spatially resolved description of the airflow field that can serve as an aerodynamic basis for assessing the subsequent transport and deposition of radioactive aerosols.

If future particle-transport simulations or experimental investigations identify significant spatial heterogeneity in aerosol deposition, the spatial relationship between the deposited radioactive material and the detector sensitive volume may become relevant to the interpretation of the measured gamma-ray signal. In such a case, equivalent amounts of deposited activity located at different positions on the filter may contribute differently to the detector response as a function of their geometric distance and relative position with respect to the detector.

This potential effect is not quantified in the present study, as neither radiation-transport calculations nor detector-response modelling is included in the CFD framework. However, the aerodynamic characterization provides a necessary upstream description of the measurement chain and establishes a basis for future multiphysics analyses coupling airflow, discrete aerosol transport, particle deposition, radioactive activity distribution, radiation transport, and detector response.

Such an integrated modelling framework would enable quantitative assessment of the complete measurement process, from air sampling and aerosol collection to the generation and interpretation of the final gamma-ray measurement.

### 4.5. Importance of the Pleated Filter Geometry

The two-dimensional analysis demonstrates that the pleated filter geometry is a significant factor governing the spatial distribution of the local airflow. The calculated mass-flux distribution shows systematic differences across the pleat, with values in the internal regions approximately 30% above the mean and values in the external regions approximately 10% below the mean. These differences indicate that the pleat geometry, in conjunction with the downstream extraction configuration, contributes directly to the development of local hydraulic non-uniformities.

This finding is particularly relevant to the design and optimization of compact radioactive aerosol monitoring systems. The pleated H13 HEPA filter should not be regarded solely as an aerosol collection element providing an increased effective filtration area; its three-dimensional geometry also determines the local flow pathways and, consequently, the spatial distribution of the aerodynamic loading across the filter surface.

Accordingly, filter geometry should be considered an explicit design parameter in the aerodynamic optimization of portable radioactive aerosol monitoring instruments. This consideration is especially important for configurations in which the radiation detector is spatially integrated within the pleated filter assembly, since the filter geometry simultaneously influences the available aerosol collection area, the local hydraulic conditions, and the spatial relationship between the collection surface and the detector.

The present results therefore highlight the importance of jointly considering filter geometry, airflow distribution, and detector arrangement during the design of compact radioactive aerosol monitoring systems. Further assessment of their coupled influence would require particle-transport and deposition modelling, together with an appropriate radiation-transport and detector-response analysis.

### 4.6. Relationship Between Flow Distribution and Filter Loading

The present CFD analysis represents the pleated H13 HEPA filter as a homogeneous porous medium characterized by spatially uniform hydraulic resistance. This formulation provides a macroscopic representation of the filter under the considered operating conditions but does not account for the progressive modification of its hydraulic properties associated with aerosol accumulation during operation.

Previous studies have demonstrated that particulate loading can increase the pressure drop across fibrous filters and alter their filtration characteristics [19,20]. Consequently, if aerosol deposition occurs non-uniformly over the filter surface, the local accumulation of particulate matter may produce spatial variations in effective permeability and hydraulic resistance. These changes could, in turn, modify the local airflow distribution, establishing a feedback mechanism between aerosol deposition and the subsequent aerodynamic field.

Conceptually, this coupling can be expressed as a sequential interaction:

Initial flow distribution → Aerosol transport → Particle deposition → Local filter loading → Permeability variation → Modified flow distribution

Such a feedback mechanism implies that the aerodynamic conditions predicted for a clean or uniformly characterized filter may evolve during operation as radioactive and non-radioactive aerosol material accumulates. The magnitude of this effect would depend on the spatial distribution and rate of particle deposition, the resulting change in filter permeability, and the corresponding redistribution of the airflow.

This coupled behaviour is not represented in the present steady-state CFD model, in which the porous-medium properties remain constant throughout the simulation. Nevertheless, it represents an important consideration for future investigations of the MARE system. A time-dependent multiphysics framework coupling airflow, particle transport, deposition, filter loading, and evolving porous-medium properties would enable assessment of how aerosol accumulation progressively modifies the hydraulic and sampling characteristics of the filter.

### 4.7. Numerical and Modelling Uncertainties and Limitations

The present CFD analysis was performed at a single operating condition of 200 m^3^ h^−1^, corresponding to the nominal flow rate of the MARE sampling subsystem. Accordingly, the results characterize the aerodynamic behaviour of the system under this specific operating condition and should not be directly extrapolated to other flow rates. A systematic analysis over a range of operating conditions would be required to quantify the dependence of the pressure drop and spatial mass-flux distribution on the imposed flow rate. This represents an important direction for future work.

#### 4.7.1. Numerical Convergence and Mesh Sensitivity

The simulations were performed using the numerical formulation and solver settings described in Section 2.5. The iterative solution was continued until the monitored flow variables exhibited stable behaviour and no relevant changes in the overall flow field were observed between successive iterations. The total flow rate was maintained at the target operating condition of 200 m^3^ h^−1^, while the pressure field and mass-flux distribution across the filter were monitored throughout the iterative solution. Particular attention was given to the pressure drop across the porous region and to the spatial distribution of mass flux at the filter outlet. The same convergence criterion was applied to both the three-dimensional and two-dimensional models.

However, a formal residual-based convergence assessment was not performed. Different convergence thresholds were not systematically evaluated, and the final residual levels and quantitative evolution of the monitored variables during the final iterations were not recorded as part of a dedicated convergence study. Therefore, although the final solutions exhibited stable behaviour of the monitored flow variables, the present study does not provide a formal quantitative estimate of numerical-convergence uncertainty for the reported pressure-drop or local mass-flux values.

Mesh resolution represents an additional source of numerical uncertainty. The three-dimensional model was discretized using 834,815 polyhedral cells, including 95,909 cells within the pleated H13 HEPA filter region. Local mesh refinement was applied around the filter, the suction pathway, and relevant geometrical transitions, while prism layers were introduced adjacent to solid surfaces. Near-wall flow was treated using the two-layer wall treatment associated with the realizable k-ε turbulence model.

Nevertheless, no systematic mesh-independence or grid-convergence study was performed. Consequently, the influence of mesh resolution on the predicted pressure drop and, particularly, on the local mass-flux distribution cannot be quantified. This limitation is especially relevant in regions characterized by strong spatial gradients, including the vicinity of the suction pathway, pleat boundaries, and geometrical transitions. Local extrema may also exhibit greater sensitivity to mesh resolution than the broader spatial distribution of the flow field.

The calculated local mass-flux values should therefore be regarded as numerical estimates obtained with the selected computational mesh rather than as quantities associated with a quantified discretization uncertainty. The interpretation consequently focuses on systematic spatial trends, such as the dominant vertical variation and the comparatively uniform circumferential distribution, rather than on isolated local extrema.

A dedicated mesh-sensitivity study using systematically refined meshes would be required to quantify the contribution of spatial discretization to the numerical uncertainty. Such an analysis should consider both integrated quantities, such as the pressure drop, and representative local quantities describing the mass-flux distribution. In the absence of this assessment, no quantitative mesh-related uncertainty interval is assigned to the present results.

#### 4.7.2. Model-to-Model Consistency

The comparison between the complete three-dimensional model and the representative two-dimensional pleat model provides a complementary assessment of model consistency. The pressure drop predicted by the three-dimensional model was 268.697 Pa, whereas the corresponding value obtained from the two-dimensional model was 262.311 Pa, yielding an absolute difference of 6.39 Pa, or approximately 2.4% relative to the three-dimensional result.

This close agreement indicates that the two modelling representations provide a comparable global hydraulic response under the considered operating condition. However, the comparison does not constitute independent validation, an accuracy estimate, or a numerical uncertainty estimate. The operating condition applied to the two-dimensional model was derived from the three-dimensional solution, and the two models differ in dimensionality, geometry, and boundary-condition formulation.

The approximately 2.4% difference should therefore not be interpreted as an uncertainty interval or as evidence that the absolute pressure-drop prediction has an accuracy of ±2.4%. Rather, it provides a model-to-model consistency check indicating that the reduced two-dimensional representation reproduces a global hydraulic response comparable to that of the complete three-dimensional model for the specific condition investigated.

#### 4.7.3. Experimental Validation

No experimental measurements of local velocity, pressure, or mass flux within the air-sampling subsystem were available for direct validation of the CFD predictions. The reference pressure-drop range of 250–300 Pa reported for the considered H13 HEPA filter under relevant operating conditions provides a consistency check for the adopted hydraulic parameterization, but it does not constitute independent experimental validation of the complete CFD model.

Accordingly, the calculated pressure drop of 268.697 Pa should be interpreted as being consistent with the adopted macroscopic hydraulic reference range rather than as independent validation of the numerical model. Experimental measurements over a range of flow rates, together with spatial measurements of pressure, velocity, or mass flux, would be required to provide a formal experimental assessment of the CFD predictions.

This limitation has been explicitly considered when interpreting the quantitative results presented in this study.

#### 4.7.4. Physical-Model Uncertainty and Porous-Medium Parameterisation

The pleated H13 HEPA filter was represented as a homogeneous porous medium using a Darcy–Forchheimer formulation. This approach describes the macroscopic hydraulic resistance of the filter without explicitly resolving its individual fibres or pore-scale structure. It is therefore appropriate for representing the global airflow and pressure loss considered in the present study, but it does not provide a fibre-resolved description of the flow through the actual filter material. Consequently, local flow features predicted within the porous domain should be interpreted as macroscopic quantities rather than as direct representations of microscopic flow structures within the fibrous medium.

An additional source of uncertainty is associated with the determination of the porous-medium parameters. The Darcy and Forchheimer coefficients were derived from the available hydraulic characterization reported in [16] and converted into the corresponding resistance formulation used in STAR-CCM+. Because the reference characterization was not performed on the specific H13 HEPA filter used in the MARE system, these coefficients should be regarded as effective macroscopic parameters for the present CFD representation rather than as independently measured intrinsic properties of the actual filter medium.

The reference pressure-drop range of 250–300 Pa was used as a consistency check of the resulting macroscopic hydraulic response and was not treated as an independent calibration dataset. Accordingly, the agreement between the predicted pressure drop and this reference range should not be interpreted as independent experimental validation.

The sensitivity of the predicted flow field to variations in the Darcy and Forchheimer coefficients was not systematically investigated. Changes in these parameters could affect both the integrated pressure loss and the local distribution of velocity and mass flux through the porous region. The resulting parameterization therefore represents a modelling assumption that contributes to the overall uncertainty of the CFD predictions but cannot be quantified from the simulations performed in the present study.

Other physical-model assumptions may also affect the quantitative results. The simulations assume steady, incompressible, constant-density airflow and employ the realizable k-ε turbulence model with the wall treatment described in Section 2.5. The sensitivity of the predicted flow field to alternative turbulence formulations, transient effects, or other physical-model assumptions was not investigated. These aspects therefore constitute additional sources of model-form uncertainty that cannot be quantified within the present study.

#### 4.7.5. Operating Conditions and Aerosol-Transport Limitations

The simulations were performed at a single flow rate of 200 m^3^ h^−1^. The sensitivity of the predicted spatial flow distribution to other operating conditions was not investigated. Since the relative contributions of viscous and inertial effects in the porous medium may vary with flow rate, the mass-flux distribution obtained at 200 m^3^ h^−1^ should not automatically be extrapolated to other operating conditions without further analysis.

Furthermore, the present CFD model describes only the continuous air phase. Aerosol particles were not explicitly tracked, and no particle-deposition, filtration-efficiency, or particle-loading model was included. The calculated mass-flux distribution therefore characterizes the aerodynamic conditions at the filter but does not constitute a quantitative prediction of aerosol deposition, particle collection efficiency, filter loading, or radioactive aerosol accumulation.

In particular, the approximately 30% increase in local mass flux identified in the internal region of the representative pleat should not be interpreted as a 30% increase in aerosol or radioactive-material deposition. The relationship between local airflow and aerosol deposition depends on particle size, inertia, Brownian diffusion, gravitational effects, particle–surface interactions, and other transport mechanisms that are not represented in the present continuous-phase model. An explicit Lagrangian particle-transport and deposition analysis would therefore be required to quantify the implications of the predicted airflow non-uniformities for aerosol collection.

#### 4.7.6. Geometrical Simplifications and Local Flow Features

The two-dimensional model represents a characteristic filter pleat and was developed to investigate local flow redistribution rather than to reproduce the complete three-dimensional geometry of the sampling system. The results obtained with this reduced model should therefore be interpreted within the scope of its simplified geometry and dimensionality.

The close agreement in global pressure drop with the three-dimensional model provides a consistency check between the two representations, but does not imply that all three-dimensional flow structures are reproduced by the two-dimensional model.

The local mass-flux distribution also exhibits small-scale irregularities associated with geometrical transitions, sharp pleat vertices, and the numerical representation of the simplified filter geometry. Such local extrema may be sensitive to both mesh resolution and geometric idealization. Accordingly, these isolated features were not considered individually when identifying the principal flow trends.

The interpretation therefore focuses on systematic spatial non-uniformities observed at the scale of the complete filter and representative pleat rather than on isolated local extrema for which a quantitative uncertainty assessment is not available.

#### 4.7.7. Implications for Detector Response

The present CFD model does not include radiation-transport calculations or a coupled detector-response model. Consequently, the results do not provide a quantitative assessment of the CeBr_3_ detector efficiency, energy response, counting statistics, or associated measurement uncertainty.

The potential relevance of the predicted airflow non-uniformities to the subsequent radiological measurement depends on the transport and deposition of radioactive aerosols and on the spatial relationship between the deposited activity and the detector sensitive volume. These effects cannot be quantified from the continuous-phase CFD results alone. Coupled aerosol-transport, deposition, radiation-transport, and detector-response analyses, supported by appropriate experimental measurements, would be required to establish a quantitative relationship between the predicted airflow distribution and the final detector response.

Overall, the absence of a formal mesh-sensitivity analysis, a dedicated residual-based convergence study, independent experimental validation, and systematic sensitivity analyses of the principal model inputs prevents the assignment of a quantitative uncertainty interval to the reported CFD results. Nevertheless, the simulations provide a consistent numerical characterization of the airflow field under the specified modelling assumptions and identify systematic spatial non-uniformities that are not captured by the global pressure-drop value alone.

The results should therefore be regarded as a macroscopic aerodynamic characterization of the MARE sampling subsystem and as a basis for subsequent experimental validation and higher-fidelity modelling. Future work should include systematic mesh-sensitivity and operating-condition studies, sensitivity analysis of the porous-medium parameters, Lagrangian particle-transport and deposition modelling, and coupled radiation-transport and detector-response calculations.

## 5. Conclusions

A CFD-based aerodynamic characterization of the air-sampling subsystem of the portable MARE system has been presented. The analysis employed complementary three-dimensional and two-dimensional porous-medium representations to investigate the macroscopic airflow behaviour through the pleated H13 HEPA aerosol collection filter. The main conclusions are summarized as follows:

- Global hydraulic response. The complete three-dimensional model predicted a pressure drop of 268.7 Pa across the pleated H13 HEPA filter at the nominal flow rate of 200 m^3^ h^−1^. This value is consistent with the reference pressure-drop range considered for the filter under the operating conditions of the sampling system. The representative two-dimensional pleat model predicted a pressure drop of 262.3 Pa, corresponding to an approximately 2.4% difference relative to the three-dimensional result. This agreement provides a model-to-model consistency check and indicates that the reduced representation reproduces a comparable global hydraulic response under the investigated operating condition.
- Spatial non-uniformity of the airflow. Despite the similar global pressure-drop predictions, the three-dimensional model demonstrated that the airflow through the filter is not spatially uniform. The circumferential distribution of mass flux is comparatively homogeneous at a given elevation, whereas the dominant spatial variation occurs along the vertical direction. The lower region of the filter exhibits increased mass flux, associated with the influence of the downstream suction configuration. These results demonstrate that the global pressure drop alone does not fully characterize the local aerodynamic conditions established within the sampling stage.
- Pleat-scale flow redistribution. The two-dimensional representative-pleat analysis provided further evidence of local flow redistribution within the filter geometry. For a mean mass flux of 1.36444 kg m^−2^ s^−1^, the internal region of the pleat exhibited values approximately 30% above the mean, whereas the external region exhibited values approximately 10% below the mean. This behaviour demonstrates that the pleated geometry and extraction configuration contribute to significant local variations in aerodynamic loading, even at the scale of an individual representative pleat.
- Implications for aerosol transport. The predicted mass-flux distribution identifies spatially heterogeneous aerodynamic conditions that may influence the subsequent transport and deposition of aerosol particles. However, the present model resolves only the continuous air phase and does not explicitly account for discrete particle trajectories, deposition mechanisms, particle-size-dependent collection, or filter loading. Consequently, the calculated mass-flux variations cannot be interpreted as direct quantitative indicators of aerosol deposition or radioactive-activity distribution.
- Relevance to radiation measurements. In the MARE configuration, the CeBr_3_ scintillation detector is located within the filter assembly, such that the spatial distribution of radioactive material accumulated on the filter may affect the radiation field reaching the detector. Establishing a quantitative relationship between the predicted airflow distribution, aerosol deposition, radioactive-activity distribution, and detector response requires additional particle-transport, deposition, radiation-transport, and detector-response modelling, supported by appropriate experimental measurements.
- Contribution to the MARE measurement chain. The air-sampling subsystem should therefore be considered an integral upstream component of the overall MARE radiation-measurement chain. The present CFD analysis provides a spatially resolved characterization of the aerodynamic conditions established during sampling and demonstrates that relevant flow non-uniformities cannot be inferred from the global pressure-drop value alone.

Overall, the principal contribution of this study is the macroscopic aerodynamic characterization of the MARE air-sampling stage rather than a direct assessment of radioactive aerosol collection efficiency or detector performance. The results establish a quantitative aerodynamic basis for subsequent investigations incorporating Lagrangian aerosol-particle transport, deposition and filter-loading effects, radiation-transport calculations, detector-response modelling, and experimental validation. Such an integrated framework would enable a more complete assessment of the coupling between sampling conditions, aerosol collection, radioactive-material distribution, and the resulting radiological measurement in the MARE system.

## 6. Future Work

The present study provides a macroscopic aerodynamic characterization of the MARE air-sampling subsystem and identifies several aspects that warrant further investigation. Future work should progressively extend the current CFD framework towards experimental validation, increased numerical robustness, explicit aerosol transport and deposition modelling, and ultimately a coupled description of the complete radiation-measurement chain.

### 6.1. Experimental Flow Validation

Experimental measurements should first be performed under operating conditions equivalent to those considered in the present CFD simulations. In particular, measurements of volumetric flow rate and pressure drop across the filter should be obtained to provide an independent assessment of the predicted global hydraulic response. Where technically feasible, spatially resolved measurements of velocity, pressure, or airflow distribution should also be conducted at representative locations within the sampling subsystem.

Such measurements would provide a quantitative basis for assessing the predictive capability of the CFD model and for identifying potential discrepancies associated with geometric simplifications, porous-medium parameterization, turbulence modelling, and boundary-condition formulation. Experimental characterization over more than one operating condition would further allow the flow-rate dependence of the numerical predictions to be evaluated.

### 6.2. Mesh Sensitivity Analysis

A systematic mesh-sensitivity study should be performed using progressively refined computational meshes. At least three representative mesh resolutions should be considered, with consistent refinement strategies applied to the principal regions of interest. The assessment should include both integral quantities, such as the pressure drop across the filter, and local quantities representative of the spatial mass-flux distribution.

Particular attention should be given to regions exhibiting strong velocity or pressure gradients, including the pleated filter, suction pathway, and major geometrical transitions. The resulting analysis would quantify the sensitivity of the predicted flow field to spatial discretization and provide a more robust basis for assessing numerical convergence and discretization uncertainty.

### 6.3. Lagrangian Particle Tracking

The continuous-phase CFD framework should subsequently be extended to include Lagrangian tracking of representative aerosol particles. Simulations covering relevant particle-size classes and, where appropriate, realistic aerosol size distributions would allow the influence of particle inertia, aerodynamic drag, gravitational settling, and Brownian motion to be investigated.

The resulting particle trajectories could be analysed in relation to the spatially resolved airflow field to determine how the predicted mass-flux non-uniformities influence particle transport through the sampling system. This approach would provide a quantitative basis for assessing particle transmission to the filter and the spatial dependence of particle collection.

### 6.4. Aerosol Deposition Modelling

The particle-transport framework should then be coupled with an appropriate deposition model to determine the spatial distribution of aerosol accumulation over the pleated filter. Such an analysis would enable the relationship between local aerodynamic conditions and particle deposition to be investigated explicitly rather than inferred from the continuous-phase mass-flux distribution.

Where sufficient information is available, the model could also incorporate the progressive accumulation of particulate material and its influence on the effective hydraulic resistance of the filter. A time-dependent treatment of filter loading would make it possible to investigate potential feedback between aerosol deposition, permeability reduction, pressure loss, and subsequent airflow redistribution.

### 6.5. Assessment of Multiple Operating Flow Rates

The present study considers a nominal sampling flow rate of 200 m^3^ h^−1^. Future simulations should investigate a range of operating conditions representative of the MARE system operating envelope. This analysis would allow the dependence of pressure drop, local mass flux, and flow non-uniformity on the imposed sampling flow rate to be quantified.

Particular attention should be given to whether the spatial trends identified at 200 m^3^ h^−1^, including the enhanced mass flux in the lower region of the filter and the redistribution observed within the representative pleat, remain consistent across the operating range or change as the relative contributions of viscous and inertial effects evolve.

### 6.6. Coupling with Radiation Transport Calculations

Once aerosol transport and deposition have been characterized, the resulting spatial distribution of radioactive material could be coupled to radiation-transport calculations and a model of the internal CeBr_3_ scintillation detector. This would provide a framework for investigating the influence of the spatial distribution of deposited activity on the radiation field incident on the detector.

Such an integrated analysis could assess the extent to which variations in the location and magnitude of deposited radioactive material influence detector efficiency, spectral response, counting statistics, and the accuracy of activity quantification. Experimental measurements would be required to evaluate the predictive capability of the coupled model.

This approach would establish a quantitative link between the upstream aerodynamic conditions, aerosol transport and deposition, radioactive-material distribution, radiation transport, and the final detector signal, while maintaining the distinction between predicted airflow non-uniformity and experimentally or numerically determined radioactive-activity distribution.

### 6.7. Advanced Modelling, System Optimization, and Data Fusion

Beyond the extensions directly derived from the present CFD framework, advanced numerical and data-driven methodologies could be investigated for system-level analysis and optimization. Recent studies have demonstrated the potential of advanced computational approaches, optimization techniques, and data-fusion methodologies for the analysis of complex engineering systems [1,2,3,4,5,6].

Within the context of the MARE system, such approaches could be used to integrate aerodynamic, aerosol-transport, deposition, radiation-transport, and detector-response models within a common computational framework. Parametric and optimization studies could then be used to investigate the influence of key design and operating variables, including filter geometry, suction-system configuration, sampling flow rate, detector position, and porous-medium characteristics.

Data-fusion approaches combining CFD predictions with experimental measurements could further support model calibration, uncertainty quantification, and system-level performance assessment. This would provide a pathway towards reduced-order or surrogate models capable of efficiently evaluating different operating and design configurations while retaining the principal physical relationships identified by the higher-fidelity simulations.

### 6.8. Integrated System-Level Framework

Collectively, these developments would extend the present aerodynamic analysis towards a comprehensive multiphysics description of the MARE measurement chain. The resulting framework could progressively link:

Air sampling → Airflow distribution → Aerosol transport → Particle deposition → Radioactive-activity distribution → Radiation transport → Detector response.

Such an integrated approach would enable the influence of sampling conditions and system geometry on the final radiological measurement to be assessed quantitatively. Importantly, the modelling framework should preserve the distinction between aerodynamic quantities calculated by the CFD model and the subsequent spatial distribution of deposited radioactive material, which requires explicit particle-transport, deposition, and radiation-transport analyses.

The proposed development would therefore provide a systematic pathway from the present macroscopic aerodynamic characterization towards a complete quantitative assessment of the MARE measurement chain, including experimental validation and uncertainty evaluation.

## Figures and Tables

**Table 1:** Distribution of computational cells among the different regions of the three-dimensional CFD domain.

Computational Region	Cells
Region 1	625,227
Filter region	95,909
Region 3	113,679
total	834,815

**Table 2:** Comparison of the pressure-drop predictions obtained from the three-dimensional and representative two-dimensional CFD models.

Model	Pressure Drop
Complete 3D model	268.70 Pa
Representative 2D model	262.31 Pa
Absolute difference	6.39 Pa
Relative difference	≈2.4%

## Data Availability

The data presented in this study are available from the corresponding author upon reasonable request. The computational model and numerical methodology are described in sufficient detail in this manuscript to facilitate reproduction of the main analysis.
